# A Mechanistic Model for Long COVID Dynamics

**DOI:** 10.3390/math11214541

**Published:** 2023-11-03

**Authors:** Jacob Derrick, Ben Patterson, Jie Bai, Jin Wang

**Affiliations:** 1 Department of Mathematics, University of Tennessee at Chattanooga, Chattanooga, TN 37403, USA;; 2 School of Mathematics and Statistics, Liaoning University, Shenyang 110036, China

**Keywords:** COVID-19, long COVID, mathematical modeling, numerical simulation, 92D30

## Abstract

Long COVID, a long-lasting disorder following an acute infection of COVID-19, represents a significant public health burden at present. In this paper, we propose a new mechanistic model based on differential equations to investigate the population dynamics of long COVID. By connecting long COVID with acute infection at the population level, our modeling framework emphasizes the interplay between COVID-19 transmission, vaccination, and long COVID dynamics. We conducted a detailed mathematical analysis of the model. We also validated the model using numerical simulation with real data from the US state of Tennessee and the UK.

## 1. Introduction

A serious consequence following infection with SARS-CoV-2 is the potential occurrence of a long-lasting disorder known as post-acute sequelae of COVID-19 (or, long COVID) [1,2]. The cause of long COVID is unclear at present, but possible contributors include persistent reservoirs of SARS-CoV-2 in certain tissues and their continued interactions with host microbiome communities, injuries to one or more organs from the acute infection, ongoing activities of primed immune cells and other immune dysregulations, clotting/coagulation issues, and dysfunctional vagus nerve signals [3–5].

A number of studies have highlighted the persistence and complications of the long COVID syndrome. For example, a cohort study followed 147 Italian patients who were hospitalized for COVID-19 and found that 87% still had symptoms 60 days after they were discharged from the hospital [6]. Another study [7] found that 76% of hospitalized COVID-19 patients (or, 1265 out of 1655) in Wuhan, China, were still experiencing symptoms 6 months after their infection. In the United States, CDC estimated that up to a third of COVID-19 cases may result in long-term symptoms [8], indicating that tens of millions of Americans diagnosed with COVID-19 may endure long-lasting consequences of the illness. The situation is further complicated by the fact that individuals who are infected with only mild symptoms, and those who are fully vaccinated but later develop breakthrough infections, can become COVID long-haulers [9–11].

In order to assess the impact of long COVID on the healthcare system and to design effective strategies for resource distribution, it is critical to quantify and predict the burden of long COVID at the population level. However, several systematic reviews and meta-analyses found that COVID long-haulers exhibited a wide variety of symptoms and that the long COVID rates are highly heterogeneous across different populations, ranging from 10% to 67% [11–15]. Such a heterogeneous pattern indicates that no single formula can be applied to every population to quantify the burden of long COVID. Instead, the evaluation and prediction of long COVID prevalence have to take into account the specific population characteristics and the epidemic/pandemic trends.

Mathematical models can overcome such challenges and help us to better quantify the population dynamics associated with long COVID. However, in contrast to the large number of mathematical, statistical, and computational models already published for COVID-19 transmission and spread (see reviews in [16–19]), very few modeling studies have been devoted to long COVID. The currently available models for long COVID are mainly based on machine learning and statistical techniques, with a focus on identifying individuals at risk of long COVID [20–25]. Their findings, though very useful, do not improve the understanding of the population dynamics of long COVID. Moreover, none of these studies have employed mechanistic models.

In this work, we propose a novel mechanistic model based on differential equations to investigate the dynamics of long COVID and its population-level prevalence. Since long COVID stems from acute infection, we will link the population dynamics of long COVID with the transmission dynamics of COVID-19. In addition, since COVID-19 vaccines have played an important role in fighting the virus, we will also incorporate the impact of vaccination in our model. Our goal is to accurately predict the prevalence of long COVID in a given population with the available data. We will implement the model in real-world applications using data from both the US and the UK.

The remainder of this paper is organized as follows. We present the formulation of our mechanistic model in Section 2 and perform a detailed mathematical analysis in Section 3. We then present data fitting and numerical simulation in Section 4. Finally, we conclude our paper with some discussion in Section 5.

## 2. Model Formulation

We used differential equations to construct our mathematical model and investigated the population dynamics of long COVID. We considered a model that involves five compartments representing the number of the following individuals: the susceptible individuals (denoted by S), the vaccinated individuals (denoted by V), the infected and infectious individuals with short-term symptoms (denoted by I), the individuals with long-term symptoms; i.e., COVID long-haulers (denoted by L), and the recovered individuals (denoted by R). Based on numerous clinical studies, the duration of infectiousness of SARS-CoV-2 is always limited to a relatively short period [26]. We, thus, assume that individuals with long COVID are not contagious.

The model is described by the following differential equations:

(1) $$\frac{dS}{dt}=\Lambda -\beta SI-(\phi +\mu )S,\;\frac{dV}{dt}=\phi S-\theta \beta VI-\mu V,\;\frac{dI}{dt}=\beta I(S+\theta V)-(\gamma +\omega +\mu )I,\;\frac{dL}{dt}=\gamma \rho I-\left(\gamma_{L}+\omega_{L}+\mu \right)L,\;\frac{dR}{dt}=\gamma (1-\rho )I+\gamma_{L}L-\mu R.$$

**Figure 1.**
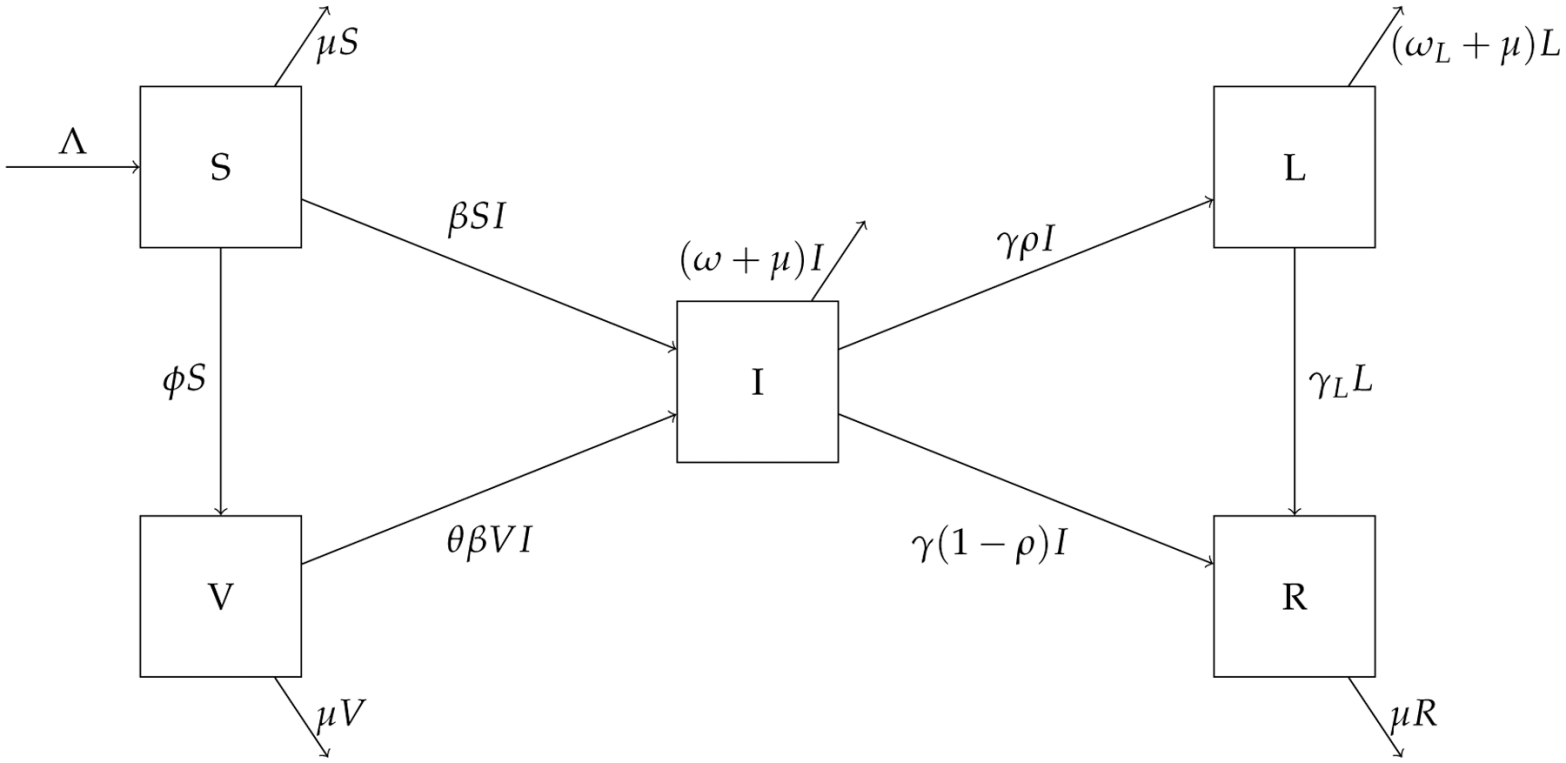
A flow chart showing movement between the compartments of the system (1).

A flow chart for this model is shown in Figure 1. The parameter Λ is the population influx rate, β is the transmission rate, μ is the natural death rate for the human hosts, and ϕ is the vaccination rate. Susceptible individuals become infected by contacting infectious individuals. In addition, susceptible individuals are vaccinated at the rate ϕ. We assume that a portion of θ(0<θ<1) in vaccinated individuals are at risk for breakthrough infections, that is, the degree of protection for the vaccines is 1-θ. Infected individuals exit the acute infection period at the rate γ; among these, a portion ρ will develop long COVID and enter the L compartment, whereas the other portion 1-ρ will truly recover from the disease and enter the R compartment. Since we are concerned with a relatively long time period, individuals in all these compartments are subject to natural mortality at an average rate μ. In addition, the parameter $\gamma_{L}$ is the rate of recovery for COVID long-haulers, and ω and $\omega_{L}$ are the disease-induced death rates in the acute infection and long COVID states, respectively.

## 3. Mathematical Analysis

System (1) has a feasible domain

(2) $$\Omega =\left\{(S,V,I,L,R)\geq 0|S\leq \frac{\Lambda }{\phi +\mu },V\leq \frac{\phi \Lambda }{\mu (\phi +\mu )},S+V+I+L+R\leq \frac{\Lambda }{\mu }\right\}.$$

It can be easily verified that Ω is positively invariant for the vector field of (1). It is also straightforward to obtain the following result:

**Theorem 1.** *System* (1) *has a unique disease-free equilibrium (DFE) in the domain* Ω

(3) $$X_{0}=\left(S_{0},V_{0},I_{0},L_{0},R_{0}\right)=\left(\frac{\Lambda }{\phi +\mu },\frac{\phi \Lambda }{\mu (\phi +\mu )},0,0,0\right).$$

The basic reproduction number of our model can be derived by the standard next-generation matrix technique [27], with the new infection matrix F and the transition matrix V computed as

$$F=\left[\begin{array}{cc} \frac{\beta \Lambda (\mu +\phi \theta )}{\mu (\phi +\mu )} & 0\\ 0 & 0 \end{array}\right],\;V=\left[\begin{array}{cc} \gamma +\omega +\mu & 0\\ -\gamma \rho & \gamma_{L}+\omega_{L}+\mu \end{array}\right].$$

The spectral radius of the next-generation matrix $FV^{-1}$ then gives the basic reproduction number of system (1):

(4) $${ℛ}_{0}=\frac{\beta \Lambda (\mu +\phi \theta )}{\mu (\phi +\mu )(\gamma +\omega +\mu )}.$$

Note that the term related to ϕθ in Equation (4) represents the risk associated with the breakthrough infection.

**Theorem 2.** *System* (1) *has a positive endemic equilibrium*

(5) $$\overset{ˆ}{X}=(\overset{ˆ}{S},\overset{ˆ}{V},\overset{ˆ}{I},\overset{ˆ}{L},\overset{ˆ}{R})$$

*in* Ω *if and only if* ${ℛ}_{0}>1$. *The endemic equilibrium is unique when it exists*.

**Proof.** At a nontrivial equilibrium of system (1), we have

(6) $$0=\Lambda -\beta SI-\left(\phi +\mu \right)S,$$

(7) 0=ϕS-θβVI-μV,

(8) 0=βIS+θβVI-(γ+ω+μ)I,

(9) $$0=\gamma \rho I-\left(\gamma_{L}+\omega_{L}+\mu \right)L,$$

(10) $$0=\gamma (1-\rho )I+\gamma_{L}L-\mu R.$$

Now, we write each equation as a function of I. From (6)–(8), we obtain

(11) $$S=\frac{\Lambda }{\beta I+\phi +\mu },$$

(12) $$V=\frac{\phi S}{\left(\theta \beta I+\mu \right)},$$

and

(13) $$V=\frac{(\gamma +\omega +\mu )-\beta S}{\beta \theta },$$

respectively. Substituting (11) into (12), we obtain

(14) $$V(I)=\frac{\phi \Lambda }{\left(\phi \beta I+\mu )(\beta I+\phi +\mu \right)}.$$

Similarly, substitution of (11) into (13) yields

(15) $$V(I)=\frac{\gamma +\omega +\mu }{\beta \theta }-\frac{\Lambda }{\theta (\beta I+\phi +\mu )}.$$

Equating (14) and (15), we obtain an equation in terms of a single variable I:

(16) $$f(I)=\frac{\gamma +\omega +\mu }{\beta \theta },$$

where the function f is defined as

(17) $$f(I):=\frac{\phi \Lambda }{\left(\phi \beta I+\mu )(\beta I+\phi +\mu \right)}+\frac{\Lambda }{\theta (\beta I+\phi +\mu )}.$$

The function f(I) is strictly decreasing for I>0, and f(I)→0 when I→∞. Hence, Equation (16) has a positive solution at $I=\overset{ˆ}{I}>0$ if and only if

(18) $$f(0)>\frac{\gamma +\omega +\mu }{\beta \theta }.$$

Through simple algebraic manipulation, it can be easily seen that Equation (18) is equivalent to ${ℛ}_{0}>1$. Clearly, the positive solution $\overset{ˆ}{I}$ is unique when ${ℛ}_{0}>1$, due to the monotonicity of the function f.

Consequently, S,V,L, and R at the positive equilibrium can all be uniquely determined from $\overset{ˆ}{I}$ based on the equations presented above, and this positive equilibrium clearly belongs to Ω. Hence, there is a unique endemic equilibrium $\overset{ˆ}{X}=(\overset{ˆ}{S},\overset{ˆ}{V},\overset{ˆ}{I},\overset{ˆ}{L},\overset{ˆ}{R})$ for system (1) if and only if ${ℛ}_{0}>1$. □

From [27], we know that the DFE $X_{0}$ is locally asymptotically stable when ${ℛ}_{0}<1$ and unstable when ${ℛ}_{0}>1$. The result below establishes the local stability for the endemic equilibrium.

**Theorem 3.** *When* ${ℛ}_{0}>1$, *the endemic equilibrium* $\overset{ˆ}{X}$ *is locally asymptotically stable*.

We provide the proof of Theorem 3 in Appendix A. In what follows, we focus on the global stability properties of the DFE and the endemic equilibrium.

**Theorem 4.** *When* ${ℛ}_{0}<1$, *the DFE* $X_{0}$ *is globally asymptotically stable in* Ω. *When* ${ℛ}_{0}>1$, *the endemic equilibrium* $\overset{ˆ}{X}$ *is globally asymptotically stable in* Ω.

**Proof.** We first consider the case ${ℛ}_{0}>1$, where the endemic equilibrium $\overset{ˆ}{X}=(\overset{ˆ}{S},\overset{ˆ}{V},\overset{ˆ}{I},\overset{ˆ}{L},\overset{ˆ}{R})$ exists and is unique. We aim to establish that all the orbits of (S,V,I) approach $\left(\overset{ˆ}{S},\overset{ˆ}{V},\overset{ˆ}{I}\right)$. To that end, we introduce the following Lyapunov function [28]:

(19) $$ℒ(S,V,I)=(S-\overset{ˆ}{S}\;lnS)+(V-\overset{ˆ}{V}\;lnV)+(I-\overset{ˆ}{I}\;lnI).$$

Taking the derivative of ℒ along the solution of system (1) gives us

(20) $$\frac{dℒ}{dt}=\left(\dot{S}-\overset{ˆ}{S}\frac{\dot{S}}{S}\right)+\left(\dot{V}-\overset{ˆ}{V}\frac{\dot{V}}{V}\right)+\left(\dot{I}-\overset{ˆ}{I}\frac{\dot{I}}{I}\right),$$

where the dot notation is used for the time derivatives of S,V, and I. We will show that $\frac{dℒ}{dt}\leq 0$. To do so, we will manipulate the three parts in Equation (20) separately. For convenience, we will denote these terms by (21)–(23). Our first term is equal to

(21) $$\left(1-\frac{\overset{ˆ}{S}}{S}\right)[\beta \overset{ˆ}{S}\overset{ˆ}{I}-\beta SI-(\phi +\mu )(S-\overset{ˆ}{S})]\;\;=\left(1-\frac{\overset{ˆ}{S}}{S}\right)[\beta (\overset{ˆ}{S}\overset{ˆ}{I}-SI)-(\phi +\mu )(S-\overset{ˆ}{S})]\;\;=\beta \left(\overset{ˆ}{S}\overset{ˆ}{I}-SI-\frac{{\overset{ˆ}{S}}^{2}\overset{ˆ}{I}}{S}+\overset{ˆ}{S}I\right)+\phi \overset{ˆ}{S}\left(2-\frac{S}{\overset{ˆ}{S}}-\frac{\overset{ˆ}{S}}{S}\right)+\mu \overset{ˆ}{S}\left(2-\frac{S}{\overset{ˆ}{S}}-\frac{\overset{ˆ}{S}}{S}\right).$$

Similarly, the second term in (20) is

(22) $$\left(1-\frac{\overset{ˆ}{V}}{V}\right)[\phi S-\theta \beta VI-\mu V-\phi \overset{ˆ}{S}+\theta \beta \overset{ˆ}{V}\overset{ˆ}{I}+\mu V]\;\;=\left(1-\frac{\overset{ˆ}{V}}{V}\right)[\phi (S-\overset{ˆ}{S})+\beta (\theta \overset{ˆ}{V}\overset{ˆ}{I}-\theta VI)+\mu (\overset{ˆ}{V}-V)]\;\;=\phi \left(S-\overset{ˆ}{S}-\frac{S\overset{ˆ}{V}}{V}+\frac{\overset{ˆ}{S}\overset{ˆ}{V}}{V}\right)+\beta \left(\theta \overset{ˆ}{V}\overset{ˆ}{I}-\theta VI-\frac{\theta {\overset{ˆ}{V}}^{2}\overset{ˆ}{I}}{V}+\theta \overset{ˆ}{V}I\right)+\mu \overset{ˆ}{V}\left(2-\frac{V}{\overset{ˆ}{V}}-\frac{\overset{ˆ}{V}}{V}\right).$$

Now, the last term of (20) is

(23) $$\left(1-\frac{\overset{ˆ}{I}}{I}\right)[\beta I(S+\theta V)-I(\gamma +\omega +\mu )-\beta \overset{ˆ}{I}(\overset{ˆ}{S}+\theta \overset{ˆ}{V})+\overset{ˆ}{I}(\gamma +\omega +\mu )]\;\;=\left(1-\frac{\overset{ˆ}{I}}{I}\right)[\beta (SI+\theta VI-\overset{ˆ}{S}\overset{ˆ}{I}-\theta \overset{ˆ}{V}\overset{ˆ}{I})]+(\gamma +\omega +\mu )(\overset{ˆ}{I}-I)\;\;=\beta \left(SI+\theta VI-\overset{ˆ}{S}\overset{ˆ}{I}-\theta \overset{ˆ}{V}\overset{ˆ}{I}-S\overset{ˆ}{I}-\theta V\overset{ˆ}{I}+\frac{\overset{ˆ}{S}{\overset{ˆ}{I}}^{2}}{I}+\frac{\theta V{\overset{ˆ}{I}}^{2}}{I}\right)+(\gamma +\omega +\mu )\overset{ˆ}{I}\left(2-\frac{I}{\overset{ˆ}{I}}-\frac{\overset{ˆ}{I}}{I}\right).$$

From here, we will begin the process of canceling out many terms and showing that all that remains are nonpositive terms. We can rewrite the following terms as

$$\left(\gamma +\omega +\mu )\overset{ˆ}{I}=\beta \overset{ˆ}{I}(\overset{ˆ}{S}+\theta \overset{ˆ}{V}\right)$$

from (8). Therefore,

$$(23)\;=\beta (SI+\theta VI-\overset{ˆ}{S}\overset{ˆ}{I}-\theta \overset{ˆ}{V}\overset{ˆ}{I})-\beta \left(S\overset{ˆ}{I}+\theta V\overset{ˆ}{I}-\frac{\overset{ˆ}{S}{\overset{ˆ}{I}}^{2}}{I}-\frac{\theta \overset{ˆ}{V}{\overset{ˆ}{I}}^{2}}{I}\right)\;\;+\beta \overset{ˆ}{S}\overset{ˆ}{I}\left(2-\frac{I}{\overset{ˆ}{I}}-\frac{\overset{ˆ}{I}}{I}\right)+\beta \theta \overset{ˆ}{V}\overset{ˆ}{I}\left(2-\frac{I}{\overset{ˆ}{I}}-\frac{\overset{ˆ}{I}}{I}\right).$$

Similarly, from (7), the terms in (22) can be rewritten as

$$\mu \overset{ˆ}{V}\left(2-\frac{V}{\overset{ˆ}{V}}-\frac{\overset{ˆ}{V}}{V}\right)=(\phi \overset{ˆ}{S}-\beta \theta \overset{ˆ}{V}\overset{ˆ}{I})\left(2-\frac{V}{\overset{ˆ}{V}}-\frac{\overset{ˆ}{V}}{V}\right),$$

giving us

$$(22)=\phi \left(S-\overset{ˆ}{S}-\frac{S\overset{ˆ}{V}}{V}+\frac{\overset{ˆ}{S}\overset{ˆ}{V}}{V}\right)+\beta \left(\theta \overset{ˆ}{V}\overset{ˆ}{I}-\theta VI-\frac{\theta {\overset{ˆ}{V}}^{2}\overset{ˆ}{I}}{V}+\theta \overset{ˆ}{V}I\right)\;+\;\phi \overset{ˆ}{S}\left(2-\frac{V}{\overset{ˆ}{V}}-\frac{\overset{ˆ}{V}}{V}\right)-\beta \theta \overset{ˆ}{V}\overset{ˆ}{I}\left(2-\frac{V}{\overset{ˆ}{V}}-\frac{\overset{ˆ}{V}}{V}\right).$$

Note that, in all of the terms of (21)–(23), there are common factors of β,μ,ϕ. We will simplify $\frac{dℒ}{dt}$ by first summing all of the terms from (21)–(23) that share one of these common factors before combining what we have left afterwards. Starting with β,

$$\beta [\overset{ˆ}{S}\overset{ˆ}{I}-SI-\frac{{\overset{ˆ}{S}}^{2}\overset{ˆ}{I}}{S}+\overset{ˆ}{S}I+\theta \overset{ˆ}{V}\overset{ˆ}{I}-\theta VI-\frac{\theta {\overset{ˆ}{V}}^{2}\overset{ˆ}{I}}{V}+\theta \overset{ˆ}{V}I-\theta \overset{ˆ}{V}\overset{ˆ}{I}\left(2-\frac{V}{\overset{ˆ}{V}}-\frac{\overset{ˆ}{V}}{V}\right)\;+\;SI+\theta VI-\overset{ˆ}{S}\overset{ˆ}{I}-\theta \overset{ˆ}{V}\overset{ˆ}{I}-S\overset{ˆ}{I}-\theta V\overset{ˆ}{I}+\frac{{\overset{ˆ}{S}\overset{ˆ}{I}}^{2}}{I}+\frac{\theta \overset{ˆ}{V}{\overset{ˆ}{I}}^{2}}{I}+\overset{ˆ}{S}\overset{ˆ}{I}\left(2-\frac{I}{\overset{ˆ}{I}}-\frac{\overset{ˆ}{I}}{I}\right)\;+\left.\theta \overset{ˆ}{V}\overset{ˆ}{I}\left(2-\frac{I}{\overset{ˆ}{I}}-\frac{\overset{ˆ}{I}}{I}\right)\right].$$

Many terms can immediately be canceled. Doing so gives

$$\beta \left[-\frac{{\overset{ˆ}{S}}^{2}\overset{ˆ}{I}}{S}+\overset{ˆ}{S}I-\frac{\theta {\overset{ˆ}{V}}^{2}\overset{ˆ}{I}}{V}+\theta \overset{ˆ}{V}I-\theta \overset{ˆ}{V}\overset{ˆ}{I}\left(2-\frac{V}{\overset{ˆ}{V}}-\frac{\overset{ˆ}{V}}{V}\right)-S\overset{ˆ}{I}-\theta V\overset{ˆ}{I}+\frac{\overset{ˆ}{S}{\overset{ˆ}{I}}^{2}}{I}+\frac{\theta \overset{ˆ}{V}{\overset{ˆ}{I}}^{2}}{I}\right.\;+\left.\overset{ˆ}{S}\overset{ˆ}{I}\left(2-\frac{I}{\overset{ˆ}{I}}-\frac{\overset{ˆ}{I}}{I}\right)+\theta \overset{ˆ}{V}\overset{ˆ}{I}\left(2-\frac{I}{\overset{ˆ}{I}}-\frac{\overset{ˆ}{I}}{I}\right)\right]\;=\beta \left[-\frac{{\overset{ˆ}{S}}^{2}\overset{ˆ}{I}}{S}+\overset{ˆ}{S}I-\frac{\theta {\overset{ˆ}{V}}^{2}\overset{ˆ}{I}}{V}+\theta \overset{ˆ}{V}I-\theta \overset{ˆ}{V}\overset{ˆ}{I}\left(2-\frac{V}{\overset{ˆ}{V}}-\frac{\overset{ˆ}{V}}{V}\right)-S\overset{ˆ}{I}-\theta V\overset{ˆ}{I}+\frac{\overset{ˆ}{S}{\overset{ˆ}{I}}^{2}}{I}+\frac{\theta \overset{ˆ}{V}{\overset{ˆ}{I}}^{2}}{I}\right.\;+\left.2\overset{ˆ}{S}\overset{ˆ}{I}-\overset{ˆ}{S}I-\frac{{\overset{ˆ}{S}\overset{ˆ}{I}}^{2}}{I}+2\theta \overset{ˆ}{V}\overset{ˆ}{I}-\theta \overset{ˆ}{V}I-\frac{\theta \overset{ˆ}{V}{\overset{ˆ}{I}}^{2}}{I}\right]\;=\beta \left[-\frac{{\overset{ˆ}{S}}^{2}\overset{ˆ}{I}}{S}-\frac{\theta {\overset{ˆ}{V}}^{2}\overset{ˆ}{I}}{V}-\theta \overset{ˆ}{V}\overset{ˆ}{I}\left(2-\frac{V}{\overset{ˆ}{V}}-\frac{\overset{ˆ}{V}}{V}\right)-S\overset{ˆ}{I}-\theta V\overset{ˆ}{I}+2\overset{ˆ}{S}\overset{ˆ}{I}+2\theta \overset{ˆ}{V}\overset{ˆ}{I}\right]\;=\beta \left[-\frac{{\overset{ˆ}{S}}^{2}\overset{ˆ}{I}}{S}-S\overset{ˆ}{I}+2\overset{ˆ}{S}\overset{ˆ}{I}-\theta \overset{ˆ}{V}\overset{ˆ}{I}\left(2-\frac{V}{\overset{ˆ}{V}}-\frac{\overset{ˆ}{V}}{V}\right)+\theta \overset{ˆ}{V}\overset{ˆ}{I}\left(2-\frac{V}{\overset{ˆ}{V}}-\frac{\overset{ˆ}{V}}{V}\right)\right]\;=\beta \left[\overset{ˆ}{S}\overset{ˆ}{I}\left(2-\frac{S}{\overset{ˆ}{S}}-\frac{\overset{ˆ}{S}}{S}\right)\right]\leq 0.$$

For terms that share a common factor of ϕ,

$$\phi \overset{ˆ}{S}\left[2-\frac{S}{\overset{ˆ}{S}}-\frac{\overset{ˆ}{S}}{S}+\frac{S}{\overset{ˆ}{S}}-1-\frac{S\overset{ˆ}{V}}{\overset{ˆ}{S}V}+\frac{\overset{ˆ}{V}}{V}+2-\frac{V}{\overset{ˆ}{V}}-\frac{\overset{ˆ}{V}}{V}\right]\;=\phi \overset{ˆ}{S}\left[3-\frac{\overset{ˆ}{S}}{S}-\frac{S\overset{ˆ}{V}}{\overset{ˆ}{S}V}-\frac{V}{\overset{ˆ}{V}}\right]\leq 0.$$

This leaves us with one remaining term with a common factor of μ:

$$\mu \overset{ˆ}{S}\left(2-\frac{S}{\overset{ˆ}{S}}-\frac{\overset{ˆ}{S}}{S}\right)\leq 0.$$

Now, by combining all of the three terms, we are left with

(24) $$\frac{dℒ}{dt}=\mu \overset{ˆ}{S}\left(2-\frac{S}{\overset{ˆ}{S}}-\frac{\overset{ˆ}{S}}{S}\right)+\beta \overset{ˆ}{S}\overset{ˆ}{I}\left(2-\frac{S}{\overset{ˆ}{S}}-\frac{\overset{ˆ}{S}}{S}\right)+\phi \overset{ˆ}{S}\left(3-\frac{\overset{ˆ}{S}}{S}-\frac{V}{\overset{ˆ}{V}}-\frac{S\overset{ˆ}{V}}{\overset{ˆ}{S}V}\right),$$

all of which are nonpositive terms since the arithmetic mean is greater than or equal to the geometric mean. Hence, $\frac{dℒ}{dt}\leq 0$ and the equality holds if and only if $\left(S,\;V,\;I)=(\overset{ˆ}{S},\overset{ˆ}{V},\;\overset{ˆ}{I}\right)$, which shows that $\left(S,V,I)\to (\overset{ˆ}{S},\overset{ˆ}{V},\overset{ˆ}{I}\right)$ for all the solution orbits. Consequently, letting $I\to \overset{ˆ}{I}$, we immediately obtain $L\to \overset{ˆ}{L}$ and $R\to \overset{ˆ}{R}$ from the last two equations of system (1). This establishes the global asymptotic stability of the endemic equilibrium $\overset{ˆ}{X}$ when ${ℛ}_{0}>1$.

When ${ℛ}_{0}<1$, the DFE $X_{0}=\left(S_{0},V_{0},I_{0},L_{0},R_{0}\right)$ is the only equilibrium of system (1). We consider the Lyapunov function

(25) $$𝒲(S,V,I)=\left(S-S_{0}\;lnS\right)+\left(V-V_{0}\;lnV\right)+I,$$

which yields

(26) $$\frac{d𝒲}{dt}=\left(\dot{S}-S_{0}\frac{\dot{S}}{S}\right)+\left(\dot{V}-V_{0}\frac{\dot{V}}{V}\right)+\dot{I}$$

along the solution of system (1). It can be easily observed that the algebraic manipulations we performed previously for $\left(\overset{ˆ}{S},\overset{ˆ}{V},\overset{ˆ}{I}\right)$ will still hold for $\left(S_{0},V_{0},0\right)$ and all the terms associated with $\overset{ˆ}{I}$ will disappear. We thus obtain

(27) $$\frac{d𝒲}{dt}=\mu S_{0}\left(2-\frac{S}{S_{0}}-\frac{S_{0}}{S}\right)+\phi S_{0}\left(3-\frac{S_{0}}{S}-\frac{V}{V_{0}}-\frac{SV_{0}}{S_{0}V}\right).$$

With similar arguments as before, we can establish the global asymptotic stability of the DFE $X_{0}$ when ${ℛ}_{0}<1$. □

These theoretical results concerning the different types of equilibria and their stability properties, with a sharp threshold at ${ℛ}_{0}=1$, are common in many epidemiological models [27,29]. Nevertheless, an important message from Theorems 2–4 is that long COVID may persist in the host population in the long run, unless COVID-19 is completely eradicated. In the next section, we will utilize a numerical simulation to implement our model for real-world applications. We fitted and simulated the model using reported data from relatively short time periods to gain insights for the population-level progression of long COVID.

## 4. Numerical Simulation

We conducted numerical simulation with real data to validate our model. In contrast to the large amount of surveillance data for COVID-19, time series data for long COVID at the population level are very rare at present. We performed two simulation studies. The first one was for the state of Tennessee in the US, where detailed data for COVID-19 cases, deaths, and vaccination coverage are available [30], but there are no population-level data available for long COVID. The second study was concerned with the UK which, as an exception, published monthly data for long COVID prevalence in the UK population through its Office for National Statistics (ONS) [31].

### 4.1. Simulation for the Tennessee State in the US

We first applied our model to the COVID-19 data for the US state of Tennessee for the 4-month period between 31 August 2021 and 30 December 2021. An estimation given by the US Census Bureau of the total population in Tennessee on 1 July 2021 gave us a value of N=6,975,218 [32]. Our time period was relatively short, so we assumed that immigration and emigration were equal, and that the natural birth rate was equal to the natural death rate, μ. We defined the natural death rate as the inverse of the life expectancy, which in 2019, before COVID-19 started in Tennessee, was 75.6 years [33]. We were then able to define the population influx rate as the product of the natural birth rate and the total population: Λ=μN. We defined γ as the recovery rate for acute infections, calculated as the reciprocal of the acute infection period reported in [34]. For ω, i.e., the disease-induced death rate for acute infections, we used the estimate provided in [35]. The breakthrough infection ratio, θ, ranged from 1% to 20% in the US [36], and we took an average θ=10% in this study. The values for these parameters can be found in Table 1.

**Figure 2.**
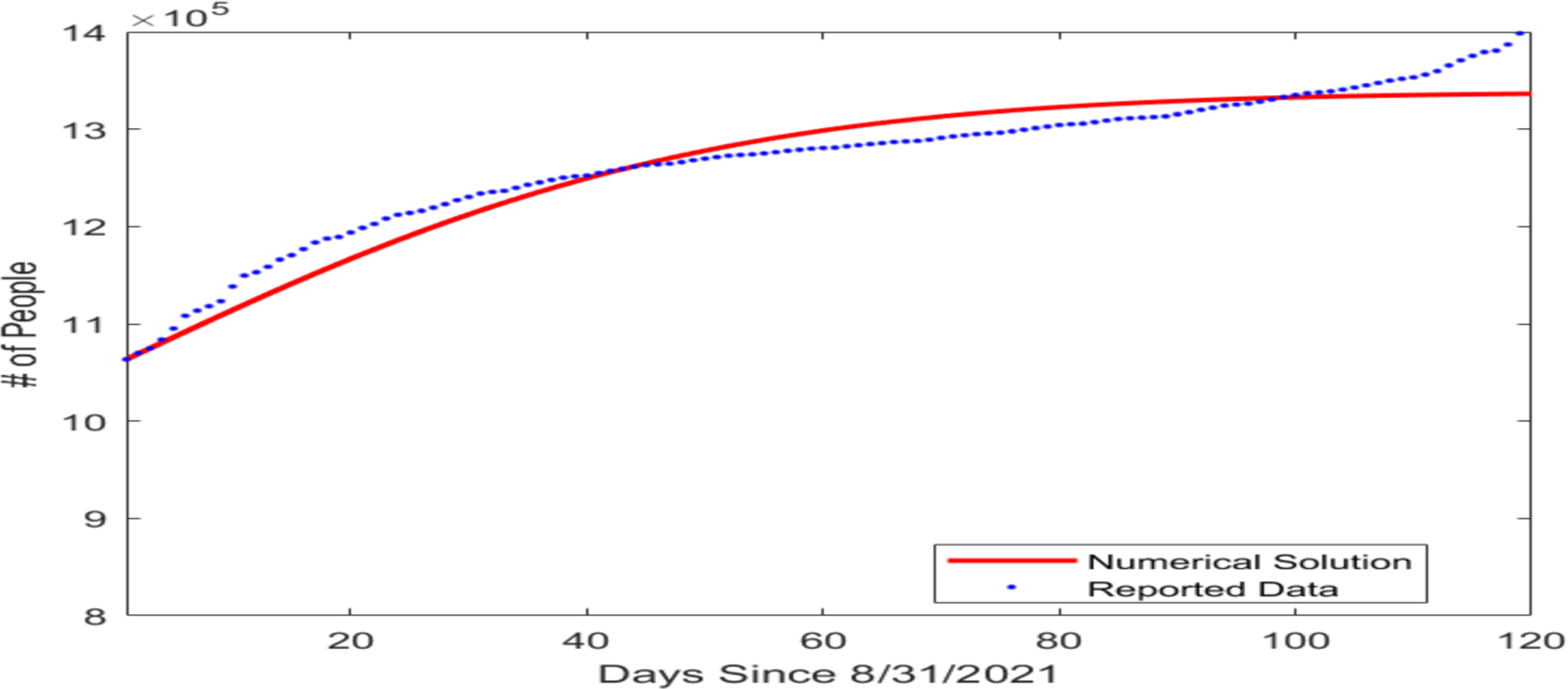
Fitting result for the number of cumulative COVID-19 cases in the US state of Tennessee beginning from 31 August 2021. The horizontal axis represents the number of days since 31 August 2021, and the vertical axis represents the number of cumulative cases.

In our model, the susceptible, vaccinated, and infected compartments form a system that is not dependent on long COVID cases or the recovered compartment. So next, we estimated the transmission rate β and the vaccination rate ϕ by fitting a simplified model consisting of only the S,V, and I compartments to the COVID-19 infection and vaccination data reported on a daily basis by the Tennessee Department of Health [30]. The initial condition for this simulation was set as (S(0),V(0),I(0))=(3,023,763,2,888,057,46,260), based on the reported data for the number of fully vaccinated individuals and infected individuals in Tennessee on 31 August 2021. The fitted parameter values are given in Table 2 and the result of the data fitting for the number of cumulative cases is shown graphically in Figure 2.

**Figure 3.**
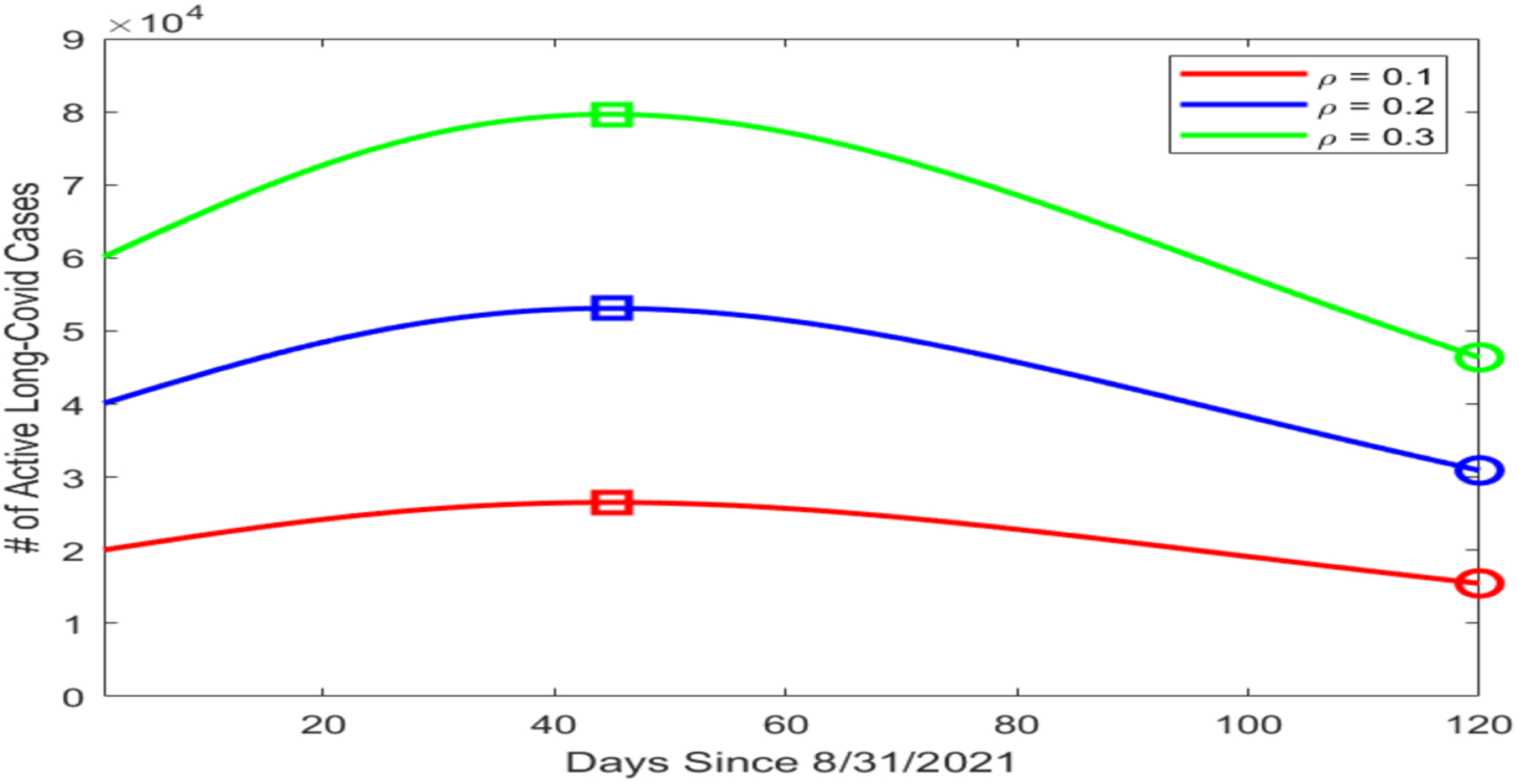
Simulation results for the number of active long COVID cases in Tennessee using different values of ρ. The horizontal axis represents the number of days since 31 August 2021, and the vertical axis represents the number of active long COVID cases. On each simulation curve, the square marks the peak and the circle marks the lowest point of the curve.

Based on our data fitting results, which included the number of active infections I in particular, we proceeded to conduct a numerical simulation for the possible prevalence levels of long COVID in Tennessee using the equation for the L compartment in system (1). We assumed that the mortality rate caused by long COVID was much lower than that caused by the acute infection, with $\omega_{L}=0.1\omega$, and that the average recovery period for long COVID was 90 days, with $\gamma_{L}=1/90$ per day. We then picked three different values for ρ, i.e., the portion of infected individuals who went on to develop long COVID, with ρ=10%, 20%, and 30%. Figure 3 displays the simulation curves for the number of active long COVID cases with the three values of ρ. The highest and lowest points on each curve are marked by a square and a circle, respectively. We observed that the peak of L ranged from about 2.5 × 10^4^ (when ρ=10%) to almost 8 × 10^4^ (when ρ=30%). Even with the minimal estimate of ρ=10%, the lowest point on the simulation curve was $L\approx 1.5\times 10^{4}$, indicating a substantial public health burden caused by long COVID.

Because long COVID data are not available for the Tennessee population, we were not able to fit the parameters relevant to long COVID dynamics and predict the progression of long COVID in Tennessee. Nevertheless, our simulation results provide possible ranges for the prevalence of long COVID in this population, which could inform the public health administration in their design of control and intervention strategies.

### 4.2. Simulation for the UK

In addition to the regular, daily reported data for COVID-19 infections, the UK Office for National Statistics (ONS) has published survey data for the prevalence of long COVID in the UK population on a monthly basis [31]. The time period covered by the long COVID data starts from 6 February 2021. The ONS data allowed us to fit our model to the number of long COVID cases. We focused our attention on fitting the L compartment in system (1), where the variable I involved in the L equation was determined by the reported number of active COVID-19 cases. We set the time from 6 February 2021 to 4 July 2022, a period about 17 months, for model fitting, and the time from 5 July 2022 to 6 December 2022, a period about 5 months, for model testing.

**Figure 4.**
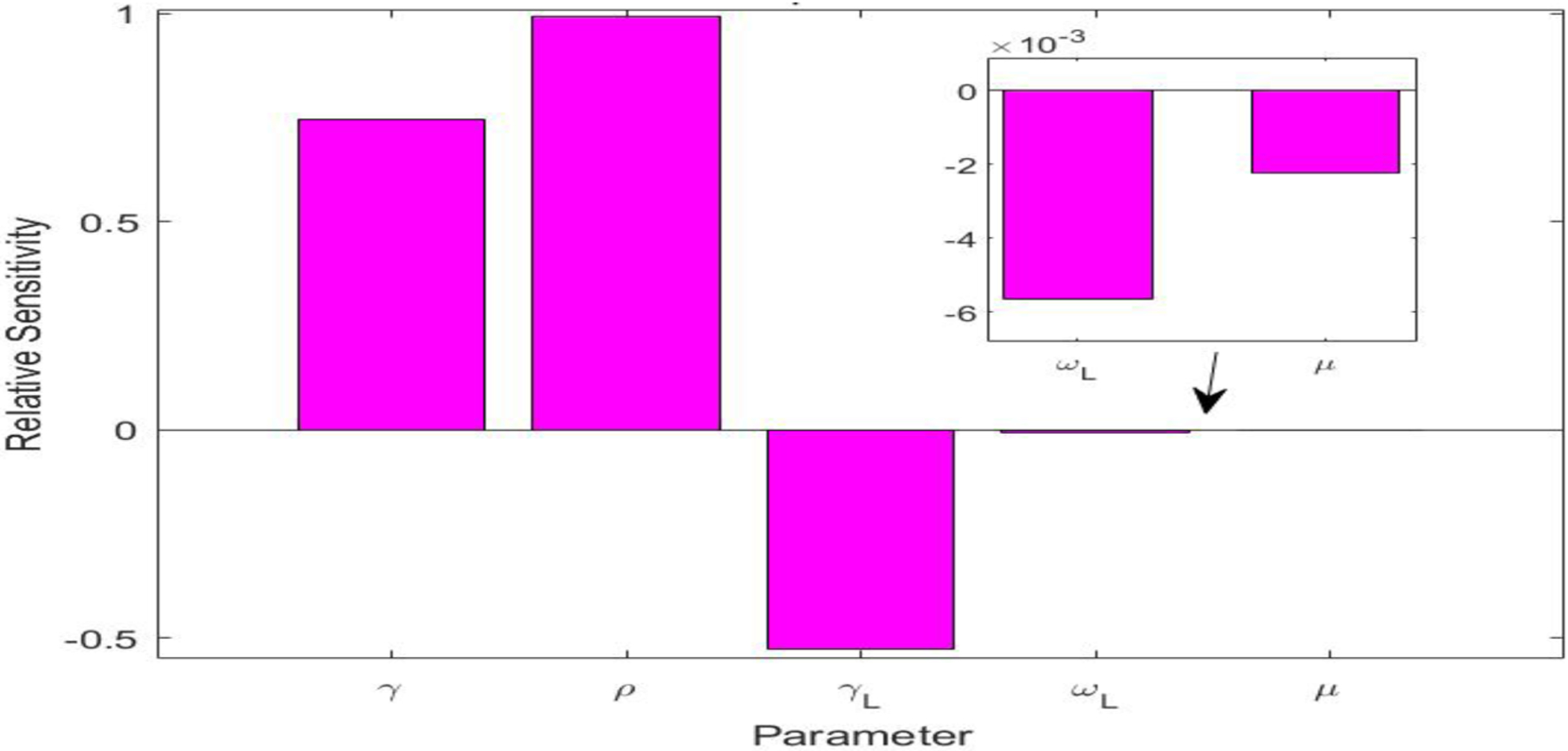
Relative sensitivities for the five parameters $\gamma ,\;\rho ,\gamma_{L},\omega_{L}$, and μ that are related to the long COVID compartment L.

The values of the parameters for the UK simulation study are listed in Table 3. There were five parameters, i.e., $\gamma ,\;\rho ,\gamma_{L},\omega_{L}$, and μ, that were involved in the L equation in system (1). We also conducted a sensitivity analysis for these five parameters in terms of the variable L, using the method from [37] for computing relative sensitivities. The results are presented in Figure 4. We observed that $\rho ,\gamma_{L}$, and γ were the three most sensitive parameters, while $\omega_{L}$ and μ had very low sensitivities. This indicates that μ and $\omega_{L}$ would have very little impact on the long COVID prevalence L. We calculated the natural death rate μ from the demographic information of the UK population [38]. We took the long COVID-induced death rate $\omega_{L}=0.0012$ per day, i.e., the same value used in the Tennessee simulation. We additionally noted that the recovery rate γ was determined by the characteristics of the acute infection. We thus set the (average) recovery period from the acute infection as $\gamma^{-1}=10$ days, based on reported values for COVID-19 in the UK [39].

We proceeded to use data fitting to estimate the two key parameters ρ and $\gamma_{L}$ associated with the long COVID prevalence. Specifically, we fitted the L equation in our model to the monthly reported long COVID data over the 17-month fitting period. Next, we conducted a numerical simulation to generate a prediction for the 5-month testing period using the parameter values estimated from the fitting period.

**Figure 5.**
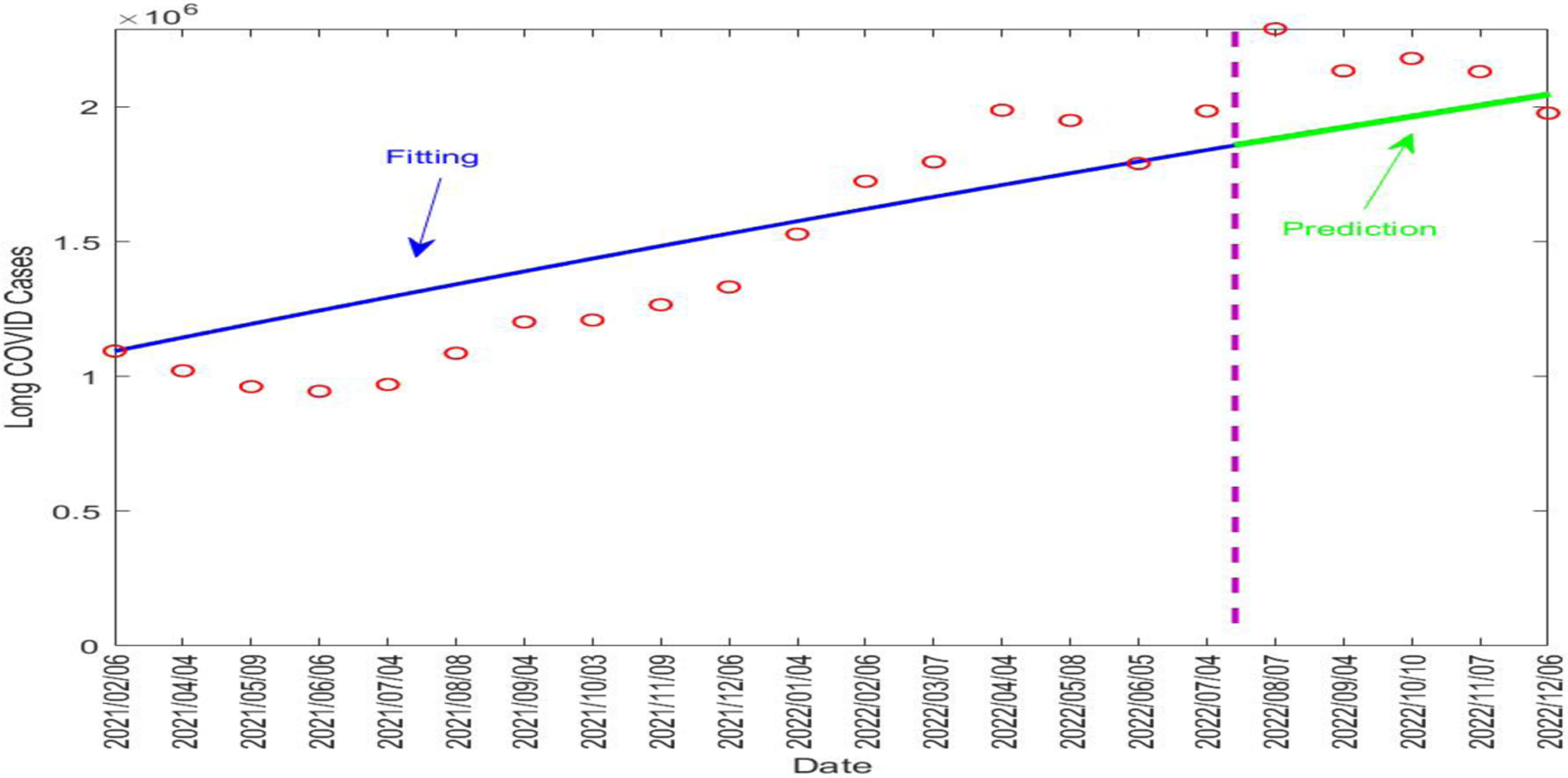
Fitting and prediction results for the long COVID cases in the UK from 6 February 2021 to 6 December 2022. The vertical dashed line in purple separates the fitting and prediction periods. The red circles represent the reported data. The blue solid line represents the fitting result and the green solid line represents the prediction result.

Table 4 lists the fitted parameter values. Figure 5 displays the fitting and prediction curves for the number of active long COVID cases in the UK, compared with the ONS data. The vertical dashed line in the figure separates the fitting and prediction periods.

Our fitting result for ρ shows that about 31.6% of the infected individuals in the UK went on to develop long COVID. The fitted value for the recovery rate of long COVID was $\gamma_{L}\approx 0.0112$ per day, indicating that long COVID would last about $1/\gamma_{L}\approx 89.3$ days on average in the UK population. This is indeed very close to the assumed value of 90 days in our simulation study for the Tennessee data. These numbers, together with the predictive capability of the model, can provide useful quantitative information to assess the burden of long COVID in the UK and to guide relevant policy developments and resource allocation.

## 5. Discussion

As stated in a recent review article, “measuring COVID-19 morbidity is an immediate priority in this pandemic” [40]. Long COVID contributes substantially to the overall COVID-19 morbidity, and quantifying the burden of long COVID at the population level is important for public health planning and policy making. In this pilot study, we present a new mechanistic model based on differential equations to investigate the population dynamics of long COVID. Our model emphasizes the interaction between COVID-19 transmission, vaccination, and long COVID dynamics. A detailed mathematical analysis was conducted and the main dynamical properties of the model were completely resolved. Furthermore, numerical simulation was carried out with real data from the US state of Tennessee and the UK to validate this modeling framework.

The two simulation studies demonstrate the utility of our modeling framework. For a place such as the US state of Tennessee, where long COVID data are not currently available, our model was able to generate useful information for the range of the long COVID prevalence at the population level. For a place such as the UK, which has published long COVID data, our model can be used to fit the prevalence and predict the future progression of long COVID. It is our hope that more population-level long COVID data will be reported in the near future, which will enable wider applications of our mechanistic model.

This work contributes to the quantitative and predictive studies of long COVID, which are emerging but still in the initial stage at present. Our findings add quantitative knowledge for the transmission of COVID-19, the progression from acute infection to long-lasting disorder, and the population-level prevalence and burden of long COVID. These results can be used to provide helpful guidelines for the public health administration to engage in science-based long COVID management and resource allocation, and for healthcare providers to target early intervention strategies and facilitate the timely recovery of long COVID patients.

The current model can be extended in several directions. For example, we may refine the model structure by adding another compartment to represent hospitalized COVID-19 patients who typically exhibit severe illness from acute infection and who are more likely to develop long COVID [6,7]. In addition, there are a number of other factors associated with COVID-19 patients that have been linked to an increased risk of long COVID, including the presence of underlying health conditions, the occurrence of multiple acute infection symptoms, old age, and a high body mass index [5,25,41]. Such factors and related clinical data may be used to improve our modeling framework.

Finally, the model proposed in this paper was built on a deterministic system of ordinary differential equations. Several other mechanistic modeling approaches, such as difference equations [42], partial differential equations [43,44], and stochastic differential equations [45,46], have been used in epidemic forecasting. These modeling techniques may also be extended to investigate the population dynamics of long COVID. It is expected that more mechanistic studies for long COVID will be generated in the near future, and it is yet to be seen which modeling technique will achieve the best performance.

## Figures and Tables

**Table 1.** Parameter values for the simulation of the Tennessee data.

Parameter	Definition	Value	Source
Λ	Population influx rate	252.780 persons per day	[32,33]
μ	Natural death rate	3.624 × 10^−5^ per day	[33]
θ	Breakthrough infection ratio	10%	[36]
$\gamma^{-1}$	Acute infection recovery period	9.5 days	[34]
ω	Death rate for acute infection	0.012 per day	[35]
β	Transmission rate	Found via data fitting	-
ϕ	Vaccination rate	Found via data fitting	-
ρ	Proportion of long COVID cases	Varied	-
$\gamma_{L}^{-1}$	Long COVID recovery period	90 days	Assumed
$\omega_{L}$	Death rate for long COVID	0.0012 per day	Assumed

**Table 2.** Parameter estimates found by fitting the Tennessee data.

Parameter	Value	95% Confidence Interval
β	4.046 × 10^−8^/person/day	(3.969 × 10^−8^, 4.123 × 10^−8^)
ϕ	0.00474/day	(0.00326, 0.00622)

**Table 3.** Parameter values for the simulation of the UK long COVID data.

Parameter	Definition	Value	Source
μ	Natural death rate	3.91 × 10^−5^ per day	[38]
$\gamma^{-1}$	Acute infection recovery period	10 days	[39]
$\omega_{L}$	Death rate for long COVID	0.0012 per day	Assumed
$\gamma_{L}^{-1}$	Long COVID recovery period	Found via data fitting	-
ρ	Proportion of long COVID cases	Found via data fitting	-

**Table 4.** Parameter estimates found by fitting the UK long COVID data.

Parameter	Value	95% Confidence Interval
$\gamma_{L}$	0.0112 per day	(0.00, 0.169)
ρ	0.316	(0.280, 1.00)

## Appendix A

**Proof of Theorem** 3. The Jacobian matrix of system (1) at the endemic equilibrium $\overset{ˆ}{X}$ is given by

$$J=\left[\begin{array}{ccccc} -\beta \overset{ˆ}{I}-\phi -\mu & 0 & -\beta \overset{ˆ}{S} & 0 & 0\\ \phi & -\theta \beta \overset{ˆ}{I}-\mu & -\theta \beta \overset{ˆ}{V} & 0 & 0\\ \beta \overset{ˆ}{I} & \theta \beta \overset{ˆ}{I} & \beta (\overset{ˆ}{S}+\theta \overset{ˆ}{V})-\gamma -\omega -\mu & 0 & 0\\ 0 & 0 & \gamma p & -\gamma_{L}-\omega_{L}-\mu & 0\\ 0 & 0 & \gamma (1-p) & \gamma_{L} & -\mu \end{array}\right].$$

We now use the characteristic equation, $det\left(\lambda I_{5}-J\right)$, to find the eigenvalues of J where $I_{5}$ is the 5 × 5 identity matrix. From elementary concepts of linear algebra, we quickly see that $det\left(\lambda I_{5}-J\right)$ is

$$\left(\lambda +\gamma_{L}+\omega_{L}+\mu \right)(\lambda +\mu )\left|\begin{array}{ccc} \lambda +\beta \overset{ˆ}{I}+\phi +\mu & 0 & \beta \overset{ˆ}{S}\\ -\phi & \lambda +\theta \beta \overset{ˆ}{I}+\mu & \theta \beta \overset{ˆ}{V}\\ -\beta \overset{ˆ}{I} & -\theta \beta \overset{ˆ}{I} & \lambda +\gamma +\omega +\mu -\beta (\overset{ˆ}{S}+\theta \overset{ˆ}{V}) \end{array}\right|.$$

Now, for the sake of clarity, let us define $a_{n,m}$ to be the corresponding matrix entry excluding any λ. Then, we have

$$det\left(\lambda I_{5}-J\right)=\left(\lambda +\gamma_{L}+\omega_{L}+\mu \right)(\lambda +\mu )\left|\begin{array}{ccc} \lambda +a_{1,1} & 0 & a_{1,3}\\ a_{2,1} & \lambda +a_{2,2} & a_{2,3}\\ a_{3,1} & a_{3,2} & \lambda +a_{3,3} \end{array}\right|\;=\;\left(\lambda +\gamma_{L}+\omega_{L}+\mu \right)(\lambda +\mu )\left(\lambda^{3}+\lambda^{2}x+\lambda y+z\right),$$

for

$$x:=a_{1,1}+a_{2,2}+a_{3,3},\;y:=a_{2,2}a_{3,3}+a_{1,1}a_{2,2}+a_{1,1}a_{3,3}-a_{2,3}a_{3,2}-a_{1,3}a_{3,1},\;z:=a_{1,1}a_{2,2}a_{3,3}+a_{1,3}a_{2,1}a_{3,2}-a_{1,3}a_{2,2}a_{3,1}-a_{1,1}a_{2,3}a_{3,2}.$$

We know that both $\left(\lambda \;+\gamma_{L}+\omega_{L}+\mu \right)$ and (λ+μ) have negative eigenvalues. Therefore, to ensure asymptotic stability, we only need to prove x,y,z>0 and xy>z to satisfy the Routh–Hurwitz criteria.

We first seek to show that x>0.

$$x\;=a_{1,1}+a_{2,2}+a_{3,3}\;=\;\beta \overset{ˆ}{I}+\phi +\theta \beta \overset{ˆ}{I}+\gamma +\omega +3\mu -\beta (\overset{ˆ}{S}+\theta \overset{ˆ}{V}).$$

This is surely positive except, potentially, for the last term. However, since we are observing this at the endemic equilibrium point $\overset{ˆ}{X}$,(6)–(10) still hold true. Thus, we quickly see from (8) that

$$\overset{ˆ}{S}=\frac{\gamma +\omega +\mu }{\beta }-\theta \overset{ˆ}{V}.$$

Therefore,

$$\beta \overset{ˆ}{I}+\phi +\theta \beta \overset{ˆ}{I}+\gamma +\omega +3\mu -\beta (\overset{ˆ}{S}+\theta \overset{ˆ}{V})\;\;=\beta \overset{ˆ}{I}+\phi +\theta \beta \overset{ˆ}{I}+\gamma +\omega +3\mu -\beta \left(\frac{\gamma +\omega +\mu }{\beta }-\theta \overset{ˆ}{V}+\theta \overset{ˆ}{V}\right)\;\;=\beta \overset{ˆ}{I}+\phi +\theta \beta \overset{ˆ}{I}+2\mu ,$$

which is positive. Hence, we have that, not only is x>0, but from our last steps, we see that $a_{3,3}=0$.

Next, we work towards proving that y>0 while noting $a_{3,3}=0$.

$$y\;=a_{2,2}a_{3,3}+a_{1,1}a_{2,2}+a_{1,1}a_{3,3}-a_{2,3}a_{3,2}-a_{1,3}a_{3,1}\;=\;a_{1,1}a_{2,2}-a_{2,3}a_{3,2}-a_{1,3}a_{3,1}\;=\;(\beta \overset{ˆ}{I}+\phi +\mu )(\theta \beta \overset{ˆ}{I}+\mu )-(\theta \beta \overset{ˆ}{V})(-\theta \beta \overset{ˆ}{I})-(\beta \overset{ˆ}{S})(-\beta \overset{ˆ}{I})\;=\;(\beta \overset{ˆ}{I}+\phi +\mu )(\theta \beta \overset{ˆ}{I}+\mu )+(\theta \beta \overset{ˆ}{V})(\theta \beta \overset{ˆ}{I})+(\beta \overset{ˆ}{S})(\beta \overset{ˆ}{I}).$$

Thus, clearly y>0.

Similarly, to show that z>0, we see that

$$z\;=a_{1,1}a_{2,2}a_{3,3}+a_{1,3}a_{2,1}a_{3,2}-a_{1,3}a_{2,2}a_{3,1}-a_{1,1}a_{2,3}a_{3,2}\;=\;a_{1,3}a_{2,1}a_{3,2}-a_{1,3}a_{2,2}a_{3,1}-a_{1,1}a_{2,3}a_{3,2}\;=\;(\beta \overset{ˆ}{S})(-\phi )(-\theta \beta \overset{ˆ}{I})-(\beta \overset{ˆ}{S})(\theta \beta \overset{ˆ}{I}+\mu )(-\beta \overset{ˆ}{I})-(\beta \overset{ˆ}{I}+\phi +\mu )(\theta \beta \overset{ˆ}{V})(-\theta \beta \overset{ˆ}{I})\;=\;(\beta \overset{ˆ}{S})(\phi )(\theta \beta \overset{ˆ}{I})+(\beta \overset{ˆ}{S})(\theta \beta \overset{ˆ}{I}+\mu )(\beta \overset{ˆ}{I})+(\beta \overset{ˆ}{I}+\phi +\mu )(\theta \beta \overset{ˆ}{V})(\theta \beta \overset{ˆ}{I}),$$

which is also clearly greater than 0.

Lastly, we show that xy>z. Note that

$$xy\;=\left(a_{1,1}+a_{2,2}\right)\left(a_{1,1}a_{2,2}-a_{2,3}a_{3,2}-a_{1,3}a_{3,1}\right)\;=\;{\left(a_{1,1}\right)}^{2}a_{2,2}+a_{1,1}{\left(a_{2,2}\right)}^{2}-a_{1,1}a_{1,3}a_{3,1}-a_{1,1}a_{2,3}a_{3,2}-a_{1,3}a_{2,2}a_{3,1}-a_{2,2}a_{2,3}a_{3,2}.$$

Here, we can note that xy and z share common terms: $-a_{1,3}a_{2,2}a_{3,1},-a_{1,1}a_{2,3}a_{3,2}$; so to prove xy>z, it suffices to show that

(A1)
$${\left(a_{1,1}\right)}^{2}a_{2,2}+a_{1,1}{\left(a_{2,2}\right)}^{2}-a_{1,1}a_{1,3}a_{3,1}-a_{2,2}a_{2,3}a_{3,2}>a_{1,3}a_{2,1}a_{3,2}.$$

Let us analyze these terms more closely. First of all, we can see that the term on the right-hand side of (A1) is

$$a_{1,3}a_{2,1}a_{3,2}=(\beta \overset{ˆ}{S})(-\phi )(\beta \overset{ˆ}{I})=\beta^{2}\overset{ˆ}{I}\overset{ˆ}{S}\phi .$$

Moreover, we see that one of the terms on the left-hand side of (A1) is

$$-a_{1,1}a_{1,3}a_{3,1}\;=-(\beta \overset{ˆ}{I}+\phi +\mu )(\beta \overset{ˆ}{S})(-\beta \overset{ˆ}{I})\;=\;\beta^{3}{\overset{ˆ}{I}}^{2}\overset{ˆ}{S}+\beta^{2}\overset{ˆ}{I}\overset{ˆ}{S}\phi +\beta^{2}\overset{ˆ}{I}\overset{ˆ}{S}\mu .$$

Therefore, we have a term that is strictly greater than what we need to prove that (A1) is true as long as none of the other terms on the left-hand side subtract away more than $\beta^{3}{\overset{ˆ}{I}}^{2}\overset{ˆ}{S}+\beta^{2}\overset{ˆ}{I}\overset{ˆ}{S}\mu$. We will see, however, that this is not a problem, and in fact, the rest of the terms will be positive additions to the left-hand side of (A1). It is easy to see that both ${\left(a_{1,1}\right)}^{2}a_{2,2}$ and $a_{1,1}{\left(a_{2,2}\right)}^{2}$ are positive values, ensuring that our inequality is true. We now look at the last value of (A1).

$$-a_{2,2}a_{2,3}a_{3,2}\;=-(\theta \beta \overset{ˆ}{I}+\mu )(\theta \beta \overset{ˆ}{V})(-\theta \beta \overset{ˆ}{V})\;=\;\theta^{3}\beta^{3}{\overset{ˆ}{I}}^{2}\overset{ˆ}{V}+\theta^{2}\beta^{2}\overset{ˆ}{I}\overset{ˆ}{V}\mu ,$$

which is another positive term. Hence, our inequality (A1) is true, proving that xy>z, Therefore, using the Routh–Hurwitz criteria, the endemic equilibrium is locally asymptotically stable when ${ℛ}_{0}>1$. □
